# Molecular Structure and Dynamics of Water on Pristine and Strained Phosphorene: Wetting and Diffusion at Nanoscale

**DOI:** 10.1038/srep38327

**Published:** 2016-12-06

**Authors:** Wei Zhang, Chao Ye, Linbi Hong, Zaixing Yang, Ruhong Zhou

**Affiliations:** 1 Institute of Quantitative Biology and Department of Physics, Zhejiang University, Hangzhou 310027, China; 2 Department of Physics, College of Sciences, China University of Mining and Technology, Xuzhou 221116, China; 3 Computational Biology Center, IBM Thomas J. Watson Research Center, Yorktown Heights, NY 10598, USA; 4 Institute of Quantitative Biology and Medicine, SRMP and RAD-X, Collaborative Innovation Center of Radiation Medicine of Jiangsu Higher Education Institutions, Soochow University, Suzhou 215123, China; 5 Department of Chemistry, Columbia University, NY 10027, USA

## Abstract

Phosphorene, a newly fabricated two-dimensional (2D) nanomaterial, has emerged as a promising material for biomedical applications with great potential. Nonetheless, understanding the wetting and diffusive properties of bio-fluids on phosphorene which are of fundamental importance to these applications remains elusive. In this work, using molecular dynamics (MD) simulations, we investigated the structural and dynamic properties of water on both pristine and strained phosphorene. Our simulations indicate that the diffusion of water molecules on the phosphorene surface is anisotropic, with strain-enhanced diffusion clearly present, which arises from strain-induced smoothing of the energy landscape. The contact angle of water droplet on phosphorene exhibits a non-monotonic variation with the transverse strain. The structure of water on transverse stretched phosphorene is demonstrated to be different from that on longitudinal stretched phosphorene. Moreover, the contact angle of water on strained phosphorene is proportional to the quotient of the longitudinal and transverse diffusion coefficients of the interfacial water. These findings thereby offer helpful insights into the mechanism of the wetting and transport of water at nanoscale, and provide a better foundation for future biomedical applications of phosphorene.

As a newly fabricated 2D nanomaterial composed of phosphorus atoms, phosphorene possesses a direct band gap which makes it a natural semiconducting nanomaterial^1^,^2^. Because of its unique electrical, optical and mechanical properties, phosphorene can be used in a wide range of biomedical applications with promising potential^3^,^4^,^5^. Notably, using a few layers of phosphorene, researchers have successfully fabricated the field-effect transistor^6^,^7^, which is a desirable device for biodetection and biosensing^8^,^9^,^10^. Simulation and experimental results showed that phosphorene in water is more stable than that in air^11^,^12^. The good stability of phosphorene in water creates the chances for phosphorene’s applications in biological fields. Compared to graphene, phosphorene is more biological-friendly due to its less disruption to proteins^4^. Moreover, as photothermal and photodynamic agents, black phosphorus nanosheets (multiple layers of phosphorene) have exhibited excellent potential in killing cancer cells^5^,^12^. Naturally, wider applications of this novel nanomaterial do require more extensive studies on its interactions with biomolecules, including the wetting and diffusive properties of bio-fluids on its surface.

The structure and dynamics of interfacial water at various nanomaterial surfaces are of fundamental importance for developing these nanomaterials’ potential applications in biomedicine and nanofluidics. The structure of water is perturbed heavily near the surfaces which have the potential to affect water diffusion^13^,^14^ and proteins’ adsorption^15^. During recent years, the wetting and diffusive properties of water in contact with two-dimensional (2D) nanomaterials, such as graphene^16^,^17^,^18^,^19^,^20^, boron-nitride sheets^16^,^21^,^22^, WS_2_ and MoS_2 _23,24, have attracted great attention. These studies have helped the promotion of potential applications of these 2D nanomaterials in both biomedicine and nanofluidics. However, the structure and dynamics of water on phosphorene, a new member of 2D nanomaterials, are still elusive to the best of our knowledge.

Another promising aspect of phosphorene is its great mechanical flexibility^25^, due to the hexagonally arranged phosphorus atoms and the subsequently formed puckered honeycomb structure inside the monolayer of phosphorene. Peng’s work demonstrated that phosphorene can withstand a uniaxial tensile strain up to 0.30^26^. Recent computational studies based on the density functional theory (DFT) showed that a single-layer phosphorene could actualize tensile strain (stretching) up to 0.54 while maintaining its original P-P bond and “two-sublayer” (non-planar) structure^27^,^28^. It has also been demonstrated that all the electrical^29^,^30^,^31^,^32^, optical^33^,^34^, thermoelectric^35^,^36^,^37^ and mechanical^27^,^30^,^38^ properties of phosphorene could be modified upon mechanical strain. The puckered structure of phosphorene brings anisotropy and negative Poisson’s ratio^27^,^35^. The strain also gives rise to phosphorene’s transition between metal and semiconductors^32^,^35^,^38^. Therefore, it is of great interest to learn if there is any change in both the structure and dynamics of water on phosphorene upon strain.

In this study, we use molecular dynamics (MD) simulations, which are widely used in the study of the interaction of (bio)molecules with nanomaterials^39^,^40^,^41^, to investigate the contact angle, diffusion coefficient, and distribution of water on both pristine and strained phosphorene. We found that the diffusion of water molecules at the surface of phosphorene is anisotropic, and strain-enhanced water diffusion is clearly present. The structure and wetting of water on transverse stretched phosphorene differs from that on the longitudinal stretched one. The contact angle of water droplet non-monotonously changes with imposed transverse strain displaying a parabola-like curve (first increases, then decreases), whereas it increases nearly linearly with imposed longitudinal strain. Also, the distribution of water near the surface of phosphorene exhibits obvious changes when imposed transverse strain increases, while the distribution of water barely changes with the longitudinal strain. Additionally, we found that the contact angle of water on strained phosphorene is proportional to the quotient of the longitudinal and transverse diffusion coefficients of the interfacial water. The dispersion energy and free energy profile of water were further calculated to interpret the above phenomena.

## Methods

MD simulations were performed to explore the wetting and diffusive properties of water on both pristine and strained phosphorene. The Gromacs package 4.5.7 42 and OPLS-AA force field^43^ were used for the simulations. The SPC/E model was used to model water molecules. Phosphorene with a dimension of 156 Å × 17 Å was chosen for this study. The bond length and bond angle of phosphorene were presented in Fig. S1 in the Supporting Information (SI). The phosphorus atoms were modeled as uncharged Lennard–Jones particles. The depth of potential well *ε*_*PP*_, cross sections *σ*_*PP*_ and bond strength constants are set at 0.400 kcal mol^−1^, 3.33 Å and 297 kcal mol^−1^ Å^2^, respectively, following a previous study based on DFT computations and experimental results^44^.

**Figure 1:**
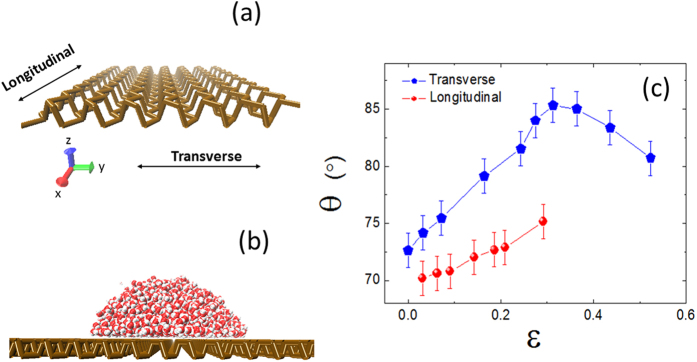
(**a**) Perspective view of transverse and longitudinal directions upon which strains were imposed. **(b)** A snapshot of water droplet on phosphorene (*ε* = 0; pristine) at the end of the MD simulation. **(c)** The water contact angle θ as a function of the transverse and longitudinal strain.

Using the umbrella pulling code of Gromacs, we applied a harmonic potential between the centers of mass of two groups of P atoms at two side of phosphorene sheet to produce the strained phosphorene, during which phosphorene was flexible. The strained phosphorene was then fixed throughout the water-phosphorene interaction simulation. Note that the strain being discussed in this study only refers to tensile strain. Phosphorene was therefore stretched along two typical directions: transverse (perpendicular to the pucker) and longitudinal (parallel to the pucker), as shown in Fig. 1(a). Phosphorene with strain ε = 0 corresponds to the pristine phosphorene. Figure S2 in the Supporting Information shows four representative configurations of strained phosphorene. The initial water droplet which was configured as a cubic consisting of 2828 molecules was set onto phosphorene under various strain conditions. During the first few nanoseconds of the simulation, the water droplet gradually converted from a cubic into a hemisphere, as illustrated in Fig. 1(b). We chose 10 morphologies of phosphorene with different degrees of transverse strain, as well as another 10 phosphorene morphologies with different degrees of longitudinal strain for our current study of the interaction with water droplet. Each phosphorene-water system was simulated for 16 ns, and the trajectory of the last 1 ns was extracted for further analysis. Following similar protocols used in our previous studies^45^,^46^,^47^,^48^,^49^,^50^,^51^, the particle-mesh Ewald method^52^ with a grid spacing of 1.2 Å was applied to simulate the long-range electrostatic interactions, and a typical cutoff of 10 Å was applied for the van der Waals interactions. We increased the z-dimension of the box (a box height of 3 times the slab height) to produce a pseudo-2D Ewald summation. Yeh and Berkowitz’s scheme for 2D Ewald summaries is another general method for systems with slab geometry^53^. All simulations were performed in an NVT ensemble at a constant temperature of 298 K by using v-rescale thermostat^54^.

The water contact angle θ was measured by fitting the time-averaged liquid-vapor interface^55^. The liquid-vapor interface is defined as the contour with half of the bulk density, while the number density of water droplet was calculated by the time-averaged spatial mesh with a grid spacing of 0.5 Å at a fixed azimuthal angle. The liquid-vapor interface was then fitted into an arc and θ was calculated as the angle of contingence at the liquid-solid interface (refer to Figure S3 in the Supporting Information).

## Results and Discussion

The Young’s modulus of phosphorene is sensitive to the direction upon which strains were imposed, due to the nanomaterial’s anisotropic nature. Figure S4 in Supporting Information illustrates the relationship between the strain at transverse and longitudinal directions and the pull force exerted along these directions. The slope of transverse strain as a function of pull force is clearly steeper than that of longitudinal strain, indicating less stiffness in phosphorene when stretched in the transverse direction. These results are consistent with previous calculations based upon first-principles density functional theory^25^,^27^.

The strains imposed upon phosphorene not only affect its electronic and mechanical properties, but also change the water contact angle on its surface. As shown in Fig. 1(c), the strains exerted in different directions have distinct effects on the water contact angle θ. An increase of the transverse strain *ε*_*T*_ causes θ to first increase, and then to decrease after the peak is reached (parabola-like curve or inverted U-shaped curve). At *ε*_*T*_ ~ 0.3, we observed the maximal contact angle θ_*max*_. However, for strains imposed upon the longitudinal direction, the relationship between θ and the longitudinal strain rate *ε*_*L*_ is clearly monotonic (roughly linear). A comparison between the contact angles of water droplet on phosphorene under different directional strains also showed an overall larger contact angles under transverse than longitudinal strain.

The microscopic contact angle has been given by the modified Young’s equation. It relates the surface tensions γ of the relevant phase (subscripts S, L, and V for solid, liquid, and vapor phase, respectively), the line tension τ and the droplet base radius *r*_*B*_ (see Fig. S3) with the contact angle θ as^55^:


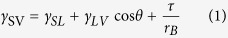


This equation implies that the contact angle is related to the size of droplet due to line tension. For the sessile water drops with linear shape, it was shown that the wetting depends on the contact line^56^. In this paper, the molecule number of a water droplet on every strained phosphorene is the same (2828 water molecules), but the base radius of the hemispherical water droplet changes with the strain, as shown in Fig. S5. This is one of the reasons why the contact angle of water droplet changes with the strain (Fig. 1c). The Equation 1 can be written as 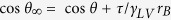, where *θ*_*∞*_ is the contact angle for macroscopic droplet. By changing the number of water molecules of droplet (the droplet size), one could derive the macroscopic contact angle *θ*_*∞*_ of droplet on phosphorene with a certain strain^57^ (see ref. 57).

In addition to the droplet size, the contact angle of a water on nanomaterials changes significantly as a function of the water-nanomaterial interaction energy^55^. If one uses a single sheet of LJ atoms in a specific lattice and tunes the interaction energy with the water molecules one would find the initial increase in contact angle due to the higher interaction with the substrate.

Along with the differences in water contact angles, the transverse and longitudinal strains also cause different deformations of the phosphorene surface. While strain in the transverse direction effectively flattens the puckers of phosphorene, longitudinal strain, on the other hand, has little effect on the bending structure of the puckers’ ring. This distinct deformation of the phosphorene monolayer leads to different interactions between the water droplet and phosphorene.

**Figure 2:**
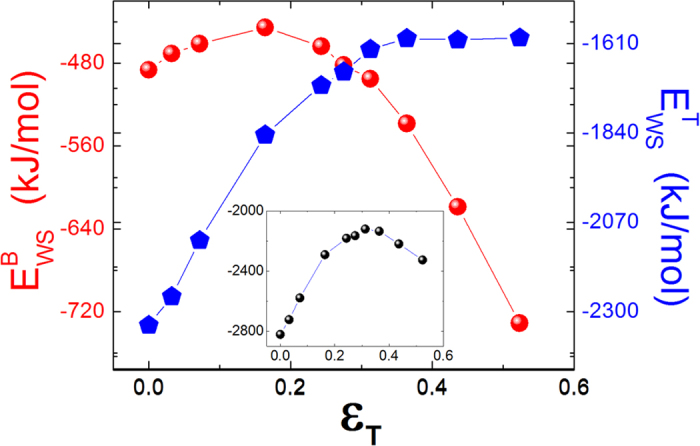
The interaction energy between the water droplet and phosphorous atoms in the bottom (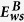 red circles) and upper (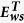 blue pentagons) surfaces of the puckered phosphorene as a function of the strain along the transverse direction. The inset shows the combined interaction energy 
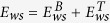
.

The contact angle θ non-monotonically changes with the transverse strain, which is primarily caused by non-monotonic variations of the interaction energy *E*_*ws*_. To analyze the influences of the transverse strain on *E*_*ws*_ in detail, we calculated the interaction energy between water droplet and phosphorous atoms in the bottom (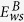) and upper (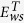) surfaces of the puckered monolayer of phosphorene, respectively (see Fig. 2). Here, 
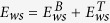
. With the increase of the transverse strain *ε*_*T*_, the density of the atoms in the upper surface decreases, thus 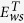 weakens remarkably (Fig. 2, blue curve), resulting in a less favorable interaction and a higher water contact angle. As for the atoms in the bottom surface, they move toward the water droplet (closer to water molecules) which is favorable for the interaction. However, its density decreases at the same time and hence is unfavorable for the interaction. When *ε*_*T*_ < ~0.16, the decrease in the density of atoms in the bottom surface dominates the interaction, and 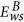 weakens slightly (see Fig. 2, red curve). When *ε*_*T*_ > ~0.16, however, the approach toward the water droplet of the atoms in the bottom surface dominates the interaction, enhancing 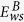 dramatically (Fig. 2, red curve). The combined effect on the interaction from both the upper and bottom surfaces atoms is thus as following, with the increase of *ε*_*T*_ the interaction energy 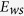 increases firstly and then decreases after *ε*_*T*_ reaches ~0.3, displaying a parabola-like curve as shown in the inset of Fig. 2. On the other hand, as mentioned above, the longitudinal strain has little effect on the bending structure of the pucker ring, thus it displays a monotonic behavior in both the interaction energy with water and the water contact angle (Fig. 1(c)).

**Figure 3:**
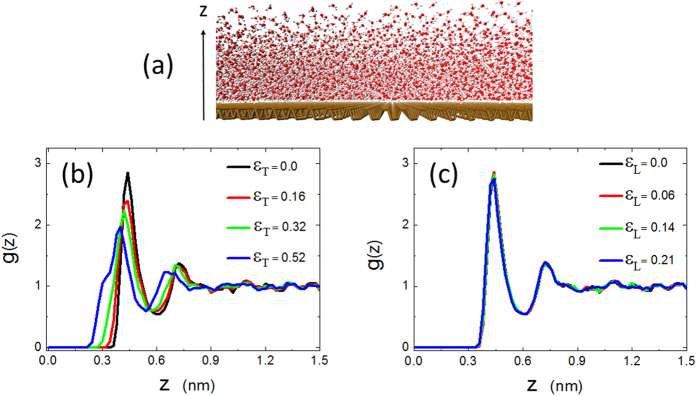
(**a**) A snapshot of the simulation system for studying the density distribution and diffusion of water molecules. **(b)** and **(c)** The density distribution function of oxygen atoms *g*(*z*) as a function of the distance z under different magnitudes of strain.

In addition to water contact angle, the strain may also affect the structure of water near phosphorene. In order to understand the structure of the interfacial water molecules, we measured the density distribution function (DDF) of the oxygen atoms along the direction normal to the surface (z axis). DDF *g*(*z*) is defined as in Equation (2):


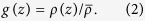


where *ρ*(*z*) is the density of oxygen atoms within a thin slice of height z parallel to the surface (the thickness of the slice is set at 0.2 Å), and 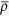 is the mean density of oxygen atoms in the bulk. Note that the zero point of z corresponds to the geometric center of phosphorene in the z-axis direction. A new and much larger thick 2D slab system consisting of 25,662 water molecules, as shown in Fig. 3(a), was used to study the density distribution and diffusion of the water molecules.

Figure 3(b) and (c) show the *g*(*z*) of the oxygen atoms near phosphorene under various transverse and longitudinal strains. Due to the dispersive interaction between the phosphorous atoms and water molecules, the structure of water near phosphorene is considerably perturbed from its bulk structure, which displays a double peak character. The double peaks of *g*(*z*) in both Fig. 3(b) and (c) indicate the two-layer structure of water in the vicinity of phosphorene, with the first peak corresponding to the first layer of water. The density of the oxygen atoms in the first water layer can be 2.8 times larger than that in the bulk. While *z* > ~1.0 *nm*, the density fluctuation disappears and the bulk density is recovered. Meanwhile, the difference between Fig. 3(b) and (c) is worth noting. In Fig. 3(b), as the transverse strain increases, both the critical position *z*_c_ where oxygen atoms appear and the maximal DDF *g*_*max*_ decrease, but the half-width of the first peak *W* increases (see Table 1). As for the longitudinal strain, it showed almost no effect on *g*(*z*), as shown in Fig. 3(c).

The above results demonstrated that the effects of transverse and longitudinal strains on the static structure of interfacial water are distinct, which can be partly attributed to the anisotropic mechanical properties of phosphorene. In the following discussion, we present the effects of the strain on the dynamic properties of interfacial water. To illustrate the dynamical properties clearly, we denote *z*_m_ and *z*_*v*_ as the z position of the first peak and valley of DDF, respectively.

In order to examine the dynamic properties of liquid on phosphorene, we begin with the self-diffusion of water molecules in the first layer. The mean squared displacement (MSD) was measured according to the following equation:


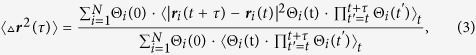


where *r*_i_(*t*) is the position of oxygen atoms of the *i* th water molecule at time *t*; *τ* is the lag time and *N* is the number of molecules included in this calculation. The angle brackets <…>_*t*_ indicate the average over the time *t*. The function of 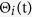 is 1 if the water molecule i belongs to the first layer at time t and is otherwise 0. MSD in Eq. 3 was evaluated only for the survived water molecule in the first layer (the position of the oxygen atom of water in the region *z*_*c*_ < *z* < *z*_*v*_). Water molecule in the first layer calculated in original time would jump out from this layer, and the continuous survival probability (CSP)^58^ decreases with the duration time (Fig. S6 in SI). The life-time of water in the first layer decreases with the transverse strain in the range (0.00–0.36). Because in this range the interaction energies between water molecules and strained phosphorene decreases with the strain.

The transverse and longitudinal mean squared displacement (MSD_T_ = 〈Δ*x*^2^(*τ*)〉 and MSD_L_ = 〈Δ*y*^2^(*τ*)〉 respectively) increases linearly with the time interval *τ*, as shown in Fig. S7. The transverse and longitudinal self-diffusion coefficients, *D*_*T*_ and *D*_*L*_, are derived from the following equations: 〈Δ*x*^2^(*τ*)〉 = 2*D*_*T*_*τ* and 〈Δ*y*^2^(*τ*)〉 = 2*D*_*T*_*τ*.

**Figure 4:**
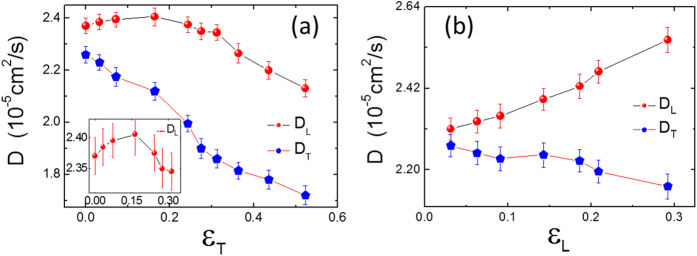
The diffusion coefficient *D* of water molecules in the first layer as a function of **(a)** the transverse and **(b)** longitudinal strain.

Figure 4 shows the transverse and longitudinal diffusion coefficients of water molecules in the first layer with various strain conditions. It is clear from the separation of the curves as shown in Fig. 4, that the diffusion of water molecules on pristine phosphorene is anisotropic, mainly due to the puckering surface morphology of phosphorene. Compared to motions in the transverse direction, a higher *D*_*L*_ coefficient indicates that it is much easier for water molecules to move along the longitudinal direction. The transverse diffusion coefficient *D*_*T*_ decreases monotonically with increased transverse strain *ε*_*T*_. The longitudinal diffusion coefficient *D*_*L*_ increases while *ε*_*T*_ is smaller than 0.16, and decreases after *ε*_*T*_ rises above 0.16, as shown in Fig. 4(a) and its inset (again, parabola-like curve or inverted U-shaped curve). The effects of longitudinal strain on the diffusion of interfacial water differ from that of transverse strain. Here, as the longitudinal strain *ε*_*L*_ increases, *D*_*L*_ also increases but *D*_*T*_ decreases. As shown in Fig. 4(b), *D*_*L*_ increases from 2.37 to 2.61 (10^−5^ cm^2^/s) in the measured range of *ε*_*L*_. In contrast, the change of *D*_*T*_ is not as significant. Clearly, the effects of *ε*_*T*_ and *ε*_*L*_ on the diffusion of interfacial water are complicated, and all of an anisotropic manner.

To rationalize the diffusion behaviors of the interfacial water, we computed the free energy profile Δ*F*(*x*, *y*) of water within the first layer (*z*_*c*_ < *z* < *z*_*v*_) (with a bin width of 5 nm in x and y-directions at the center of phosphorene sheet) with the equation:


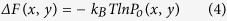


**Figure 5:**
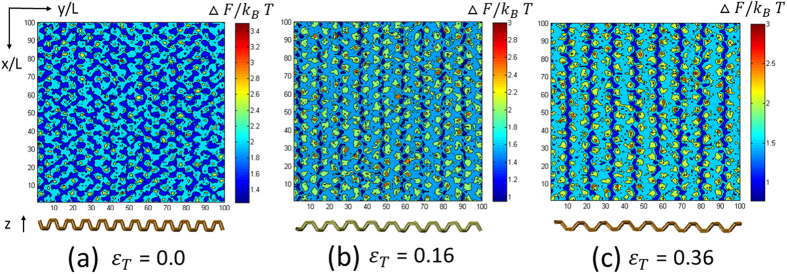
Free energy profile of water within the first layer Δ*F*(*x*, *y*) scaled by *k*_*B*_*T* for three values of *ε*_*T*_. Here, the scaling parameter L = 0.05 nm.

Here, *P*_*o*_(*x, y*) is the spatial probability distribution function of the oxygen atoms of water within the first layer at coordinate (*x, y*). This approach has been previously applied to examine the friction of water on graphene and boron nitride^22^, as well as the free energy landscape of protein folding^59^,^60^,^61^. In Fig. 5, we present Δ*F*(*x, y*) as scaled by 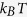 under different transverse strains. The free energy profile Δ*F*(*x, y*) obviously exhibits a “grooved morphology”, mimicking the phosphorene surface, which is a clear indication of anisotropy. The minimal free energy regions appear in the grooves of phosphorene. The variation of the free energy profile with the strain arises from the change of the structure of phosphorene. The hopping mechanism of interfacial water would depend upon free energy barrier. A smooth free energy profile is beneficial to the diffusion of the interfacial water. The smoother the free energy profile is (corresponding to smaller energy barrier), the larger the diffusion coefficient of interfacial water becomes.

For the pristine phosphorene (*ε*_*T*_ = 0 and *ε*_*L*_ = 0), the free energy profile Δ*F*(*x, y*) shows clear “zigzag” patterns in the grooved region (see Fig. 5(a)), similar to the so-called “swallow gird”. The maximal energy barrier for water molecules to translate along the longitudinal direction is about 1.5 *k*_*B*_*T*, while it is ~1.7 *k*_*B*_*T* to move in the transverse direction. Thus, it is more difficult for interfacial water molecules to move in the transverse than in the longitudinal direction. As a result, the diffusion coefficient of interfacial water along the longitudinal direction is larger than that along the transverse direction.

### Rationalizing the effects of the transverse strain *ε*_*T*_ on the transverse diffusion coefficient *D*_*T*_

The strain *ε*_*T*_ acts to broaden the interval of free energy ribbons (low-energy region along the groove) which corresponds to the groove width of phosphorene. The broadening of free energy ribbon interval constrains the diffusion of water molecules along the transverse direction, since it introduces more difficulty in water molecules’ crossing of broadened energy barrier. Meanwhile, the strain *ε*_*T*_ increases the mean energy barrier for water molecules to move in the transverse direction, thus hindering the motion of water molecules along the transverse direction. Consequently, the transverse diffusion coefficient *D*_*T*_ decreases as *ε*_*T*_ increases, as shown in Fig. 4(a).

### Rationalizing the effects of the transverse strain *ε*_*T*_ on the longitudinal diffusion coefficient *D*_*T*_

With the increase of strain *ε*_*T*_, the “zigzag” pattern gradually disappears in general and the free energy ribbons becomes smooth in the longitudinal direction (the low-energy region inter-connects, see Fig. 5(b) and (c)), which is favorable for water molecules to diffuse in the longitudinal direction. Thus, the longitudinal diffusion coefficient *D*_*L*_ increases with the increase of *ε*_*T*_, up to 0.16, as shown in the inset of Fig. 4(a). With further increase of *ε*_*T*_ to be larger than 0.16, the influence of phosphorus atoms at the bottom layer of the groove begins to dominate as mentioned above, which affects water’s diffusion as well. As *ε*_*T*_ increases above 0.16, *D*_*L*_ decreases, because of the increased attraction from phosphorus atoms at the bottom of the groove (see Fig. 2).

### Rationalizing the effects of the longitudinal strain *ε*_*T*_ on the longitudinal and transverse diffusion coefficients

When phosphorene is under longitudinal strain, the variations of the free energy profile Δ*F*(*x, y*) is different from those under transverse strain (see Fig. S8 in Supporting Information). The longitudinal strain *ε*_*L*_ obviously attenuates the “zigzag” pattern of the free energy ribbon, which accounts for the enhancement of *D*_*L*_ as *ε*_*L*_ increases. However, unlike the effect of *ε*_*T*_, the longitudinal strain *ε*_*L*_ only narrows the groove width, while shows little effect on its depth. In other words, it increases the mean height of energy barrier (see Fig. S9) but decreases its width. The increased energy barrier constrains the diffusion of water molecules along the transverse direction. On the other hand, the decrease of the width of energy barrier is in favor of water diffusion, thus an increased *D*_*T*_. Under the combined influences of these two factors (the increased height and decreased width of energy barrier), as *ε*_*L*_ increases, the transverse diffusion coefficient *D*_*T*_ decreases slightly.

Though the longitudinal strain *ε*_*L*_ obviously smoothes the free energy ribbon and increases the longitudinal diffusion coefficient of interfacial water, it has almost no effects on the density distribution function (DDF) *g*(*z*) (see Fig. 3(c)). The DDF *g*(*z*), however, obviously changes with the transverse strain *ε*_*T*_ (Fig. 3(b)), which is caused by the flattening of the puckered surface of phosphorene. The flattening of the surface of phosphorene leads to the decrease of the critical position *z*_*c*_. The increase of the half-width W and the decrease of the height *g*_*max*_ are related to the energy profile of single water molecule on phosphorene surface as a function of distance from surface (see Fig. S10). As shown in Fig. S10, energy profile curve are similar to LJ potential and has a minimum value. With the increase of the transverse strain, the minimum energy value and the half-width of the energy valley increase. Consequently, DDF *g*(*z*) is related not only to the dispersion energy between water and phosphorene, but also to the distribution of water molecules on phosphorene’s surface.

**Figure 6:**
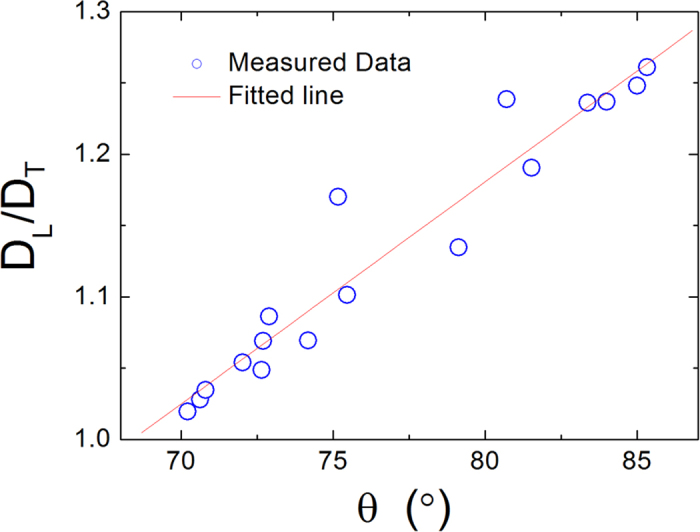
The quotient of *D*_*L*_/*D*_*T*_ as a function of the contact angle θ of water droplets. The fitting line has the form: *D*_*L*_/*D*_*T*_ = θ ∙ 0.016(^0^)^−1^ − 0.06.

Interestingly, the quotient *D*_*L*_/*D*_*T*_, instead of *D*_*L*_ or *D*_*T*_ alone, exhibits a roughly linear relationship with the contact angle θ of water droplet, as shown in Fig. 6. The variation of the width and depth of the groove of phosphorene caused by the strain directly affects the interaction energy *E*_*ws*_ and the free energy profile Δ*F*(*x, y*). The contact angle θ and the diffusion coefficient of water molecules in the first layer, on the other hand, are mainly determined by the interaction energy *E*_*ws*_ and free energy profile Δ*F*(*x, y*). The free energy profile Δ*F*(*x, y*) jointly affects the transverse and longitudinal diffusion of water molecules. In this sense, the contact angle θ should be related to the diffusion of water molecules along transverse and longitudinal directions. However at present, we have not yet found a quantitative interpretation regarding the relationship between the ratio *D*_*L*_/*D*_*T*_ and water contact angle θ, which needs further investigation and experimental validation.

Changing the pattern of the puckering surface of phosphorene by the strain could effectively enhance or attenuate the diffusion of interfacial water molecules, which might provide insight for controlling/designing the motion of interfacial molecules. By controlling the strain, one could construct continuous diffusion (or wetting) gradient, which is of interest in artificial microscopic walk^62^,^63^. The anisotropic diffusion of water molecules near phosphorene surface may also affect phosphorene’s motion in complex biological systems, which is of importance for phosphorene’s potential biomedical applications, such as localized bioprobes and drug delivery.

## Conclusions

In this study, we investigated the wetting and diffusive properties of water near both pristine and strained phosphorene with MD simulations. It was found that the pristine phosphorene is of weak hydrophobicity, with a water contact angle of ~72°. As for the interactions between water droplet and phosphorene under different strains (stretchings), we discovered that the water contact angle θ displays a parabola-like curve with the transverse strain (first increases, then decreases, as the transverse strain reaches a critical threshold of 0.3). However, for the longitudinal strain, the contact angle θ increases monotonically as the strain increases. The changes in water contact angle are mainly determined by the interaction energy between the water droplet and phosphorene. The structure of the interfacial water dramatically changes with the transverse strain *ε*_*T*_, but the longitudinal strain *ε*_*L*_ has almost no effect on water’s structure.

While the diffusion of water molecules near pristine phosphorene surface is anisotropic, the longitudinal diffusion coefficient *D*_*L*_ is larger than that (*D*_*T*_) in the transverse direction. As the transverse strain *ε*_*T*_ increases, *D*_*T*_ decreases monotonically, while *D*_*L*_ exhibits a parabola-like curve (or inverted U-shaped curve). The longitudinal strain *ε*_*L*_, on the other hand, causes *D*_*L*_ to increase, and *D*_*T*_ to decrease monotonically. We also calculated the free energy profile ΔF(*x, y*) to determine the main cause of variations in the diffusion of water molecules near phosphorene. It was shown that the smoothing of the energy landscape enhances *D*_*L*_, and the increased energy barrier causes *D*_*T*_ to decrease. Last but not least, we found that interestingly the quotient *D*_*L*_/*D*_*T*_ is positively correlated with the contact angle θ.

As a novel 2D nanomaterial, phosphorene has the potential to be widely used in biomedical applications in the near future. Therefore it is necessary to investigate their interactions with various bio-fluids, including the wetting and diffusive properties of water on both pristine and strained phosphorene. Our study should help with understanding and manipulating the wetting and diffusive properties of liquid on phosphorene, which is critical for phosphorene’s application in the fields of biomedicine and nanofluidics.

## Additional Information

**How to cite this article**: Zhang, W. *et al*. Molecular Structure and Dynamics of Water on Pristine and Strained Phosphorene: Wetting and Diffusion at Nanoscale. *Sci. Rep.* **6**, 38327; doi: 10.1038/srep38327 (2016).

**Publisher's note:** Springer Nature remains neutral with regard to jurisdictional claims in published maps and institutional affiliations.

## Supplementary Material

Supplementary Information

## Figures and Tables

**Table 1:** The critical position *z*_*C*_, the height *g*_*max*_ and half-width W of the first peak of the density distribution function (DDF) of oxygen atoms corresponding to various values of the transverse strain *ε*_T_.

ε_*T*_	*z*_*c*_ (nm)	g_*max*_	W (nm)
0	0.36	2.85	0.08
0.03	0.35	2.68	0.10
0.07	0.34	2.64	0.10
0.16	0.32	2.38	0.10
0.24	0.30	2.26	0.12
0.28	0.30	2.21	0.12
0.31	0.28	2.24	0.12
0.36	0.25	2.14	0.14
0.43	0.24	2.08	0.15
0.52	0.22	1.97	0.18
