# Supplementary material for: Molecular Structure and Dynamics of Water on Pristine and Strained Phosphorene: Wetting and Diffusion at Nanoscale
Source: Sci Rep. 2016 Dec 6;6:38327. doi: 10.1038/srep38327 (PMC5138611; doi:10.1038/srep38327)
Supplement: Supplementary Information [file srep38327-s1.pdf]

Supporting Information:

Molecule structure and dynamics of water on pristine and strained phosphorene: Wetting and diffusion at nanoscale

Wei Zhang [1,2,*], Chao Ye [1,*], Linbi Hong [3], Zaixing Yang [4] and Ruhong Zhou [1,3,5]

[1] Institute of Quantitative Biology and Department of Physics, Zhejiang University, Hangzhou 310027, China

[2] Department of Physics, College of Sciences, China University of Mining and Technology, Xuzhou 221116, China

[3] Computational Biology Center, IBM Thomas J. Watson Research Center, Yorktown Heights, NY 10598, USA

[4] Institute of Quantitative Biology and Medicine, SRMP and RAD-X, Collaborative Innovation Center of Radiation Medicine of Jiangsu Higher Education Institutions, Soochow University, Suzhou 215123, China

[5] Department of Chemistry, Columbia University, NY 10027, USA

Figure S1. The bond length and bond angle of phosphorene

Figure S2. The configuration of phosphorene under longitudinal and transverse strain. **(a)** Structure without deformation (ε = 0) **(b)** Tension with $\varepsilon_L = 0.14$ along longitudinal direction. **(c)** and **(d)** Tension with $\varepsilon_T = 0.16$ and $\varepsilon_T = 0.52$ along transverse direction. Top and side views are displayed in all panels.

Figure S3. Determination of the contact angle θ of water droplet by fitting the time-averaged liquid/vapor interface. The scaling parameter $\mathrm{L} = 0.05\,\mathrm{nm}$. The density profile of water droplet on phosphorene was exhibited by the colored contour line.

Figure S4. The transverse and longitudinal strain as a function of the force exerted on phosphorene along the corresponding direction.

Figure S5. The droplet water size r_B as a function of the strain along (blue pentagons) transverse and (red circles) longitudinal direction.

Figure S6. The continuous survival probability (CSP) as a function of the time for different transverse and longitudinal strain.

Figure S7. The transverse and longitudinal MSD as a function of the time for water molecules in the first layer above phosphorene with the strain $\varepsilon_T = 0.32$.

Figure S8. Free energy profile of water within the first layer $\Delta F(x, y)$ scaled by $k_B T$ for three values of ε_L. The scaling parameter $L = 0.05$ nm.

Figure S9. The free energy difference between the minima and maxima as a function of the strain along longitudinal direction.

Figure S10. The profile of single water molecule on phosphorene surface with various strain as a function of distance from surface.

The continuous survival probability (CSP) is defined as follows [1]:

$$\mathrm{CSP}(\tau) = \frac{\sum_{i=1}^{N} \Theta_i\,(0) \cdot \prod_{t'=0}^{\tau} \Theta_i\,(t')}{\sum_{i=1}^{N} \Theta_i\,(0)}, \qquad (S1)$$

where the function of $\Theta_i(t)$ is 1 if the water molecule i belongs to the first water layer (z_c<z<z_v) at time t and is otherwise 0.

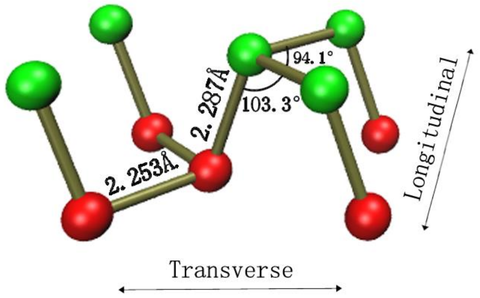

Figure S1. The bond length and bond angle of phosphorene

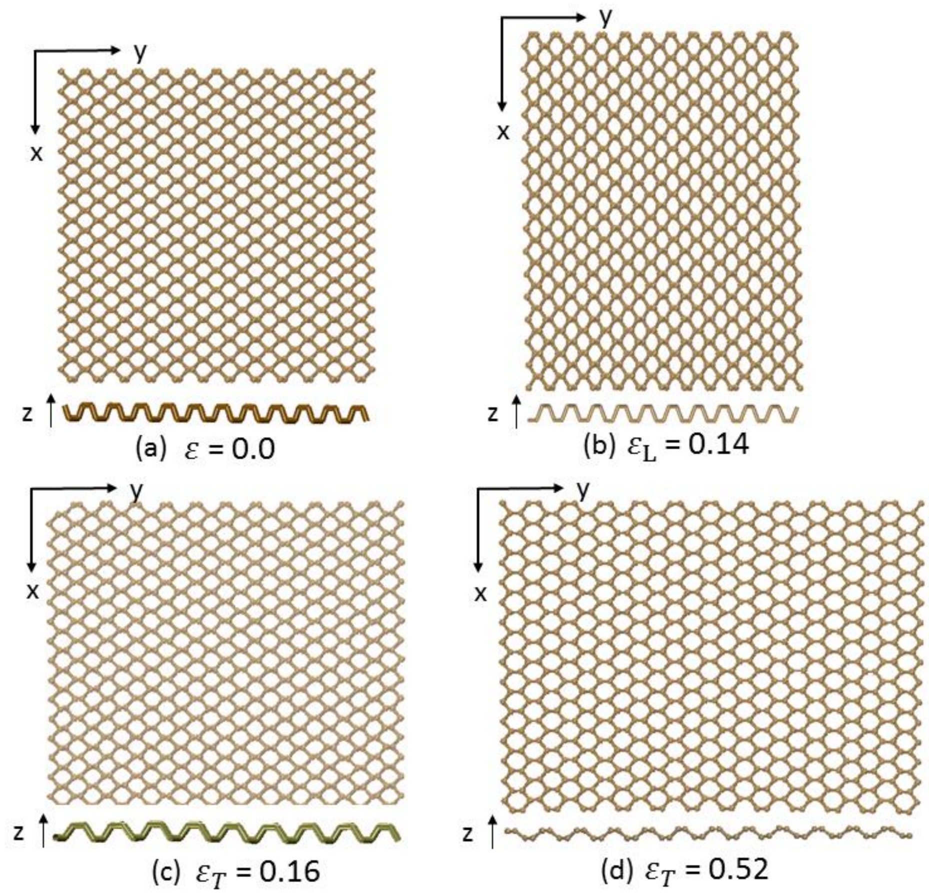

Figure S2. The configuration of phosphorene under longitudinal and transverse strain. **(a)** Structure without deformation ($\varepsilon = 0$) **(b)** Tension with $\varepsilon_L = 0.14$ along longitudinal direction. **(c)** and **(d)** Tension with $\varepsilon_T = 0.16$ and $\varepsilon_T = 0.52$ along transverse direction. Top and side views are displayed in all panels.

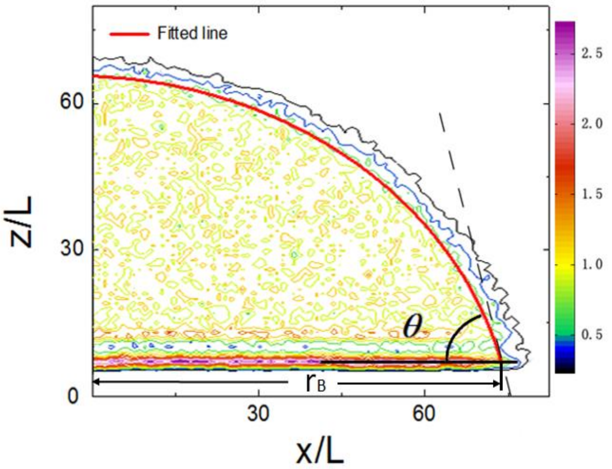

Figure S3. Determination of the contact angle θ of water droplet by fitting the time-averaged liquid/vapor interface. The scaling parameter $L = 0.05$ nm. The density profile of water droplet on phosphorene was exhibited by the colored contour line. The red line is the fitted liquid/vapor interface. The horizontal black line corresponds to the position of z_m.

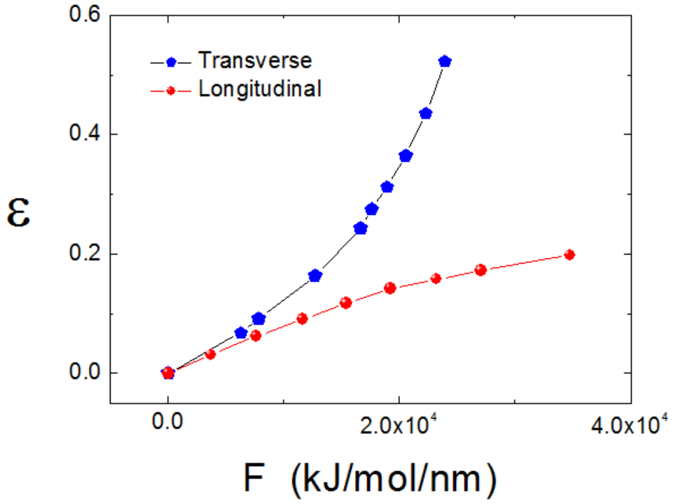

Figure S4. The transverse and longitudinal strain as a function of the force exerted on phosphorene along the corresponding direction.

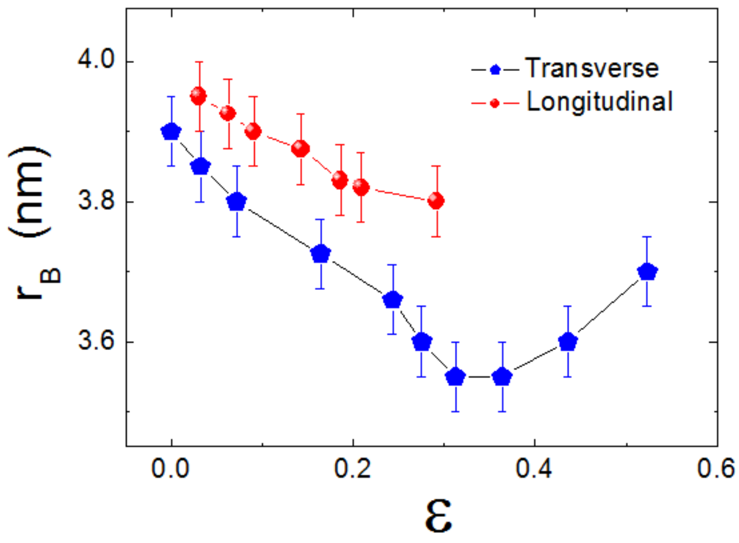

Figure S5. The droplet water size r_B as a function of the strain along (blue pentagons) transverse and (red circles) longitudinal direction.

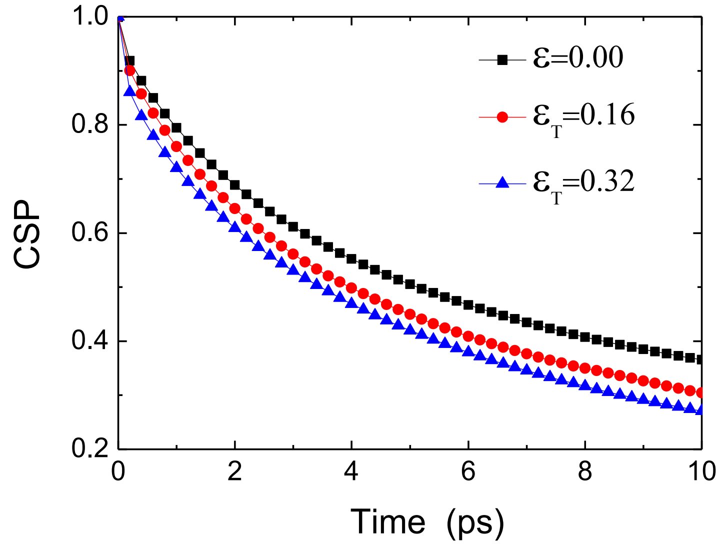

Figure S6. The continuous survival probability (CSP) as a function of the time for different transverse and longitudinal strain.

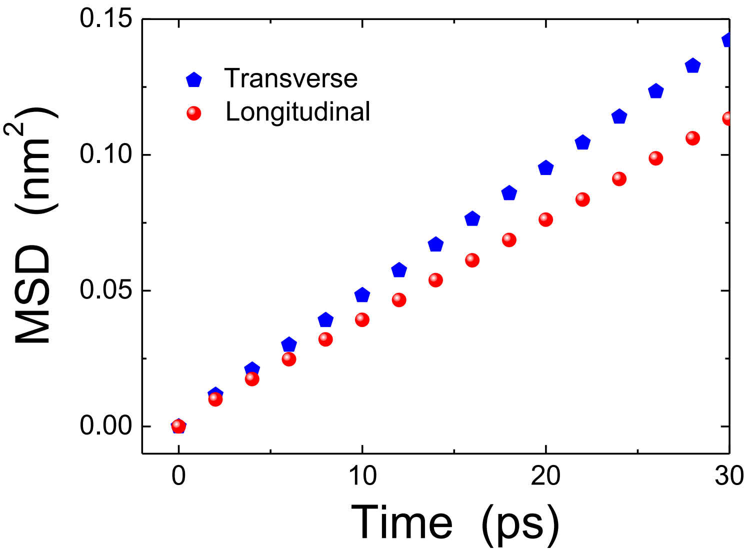

Figure S7. The transverse and longitudinal MSD as a function of the time for water molecules in the first layer above phosphorene with the strain $\varepsilon_T = 0.32$.

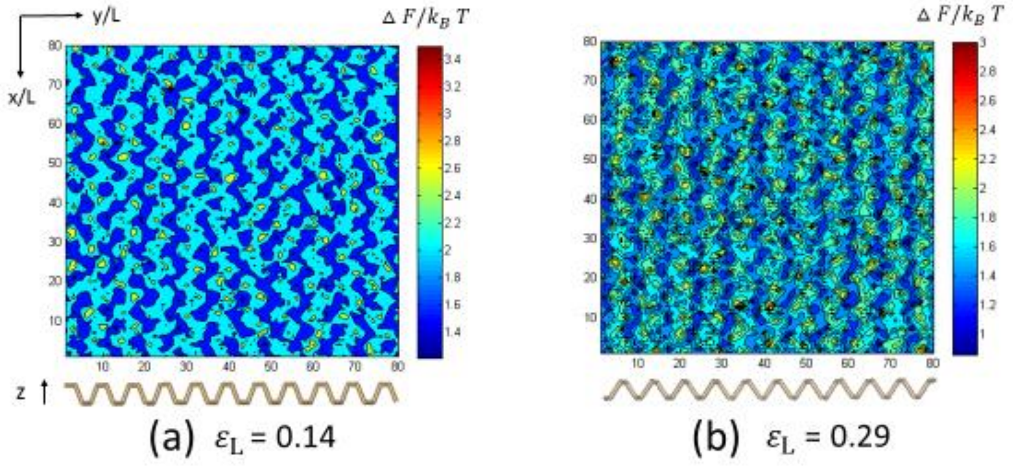

Figure S8. Free energy profile of water within the first layer $\Delta F(x, y)$ scaled by $k_B T$ for three values of ε_L. The scaling parameter $L = 0.05$ nm.

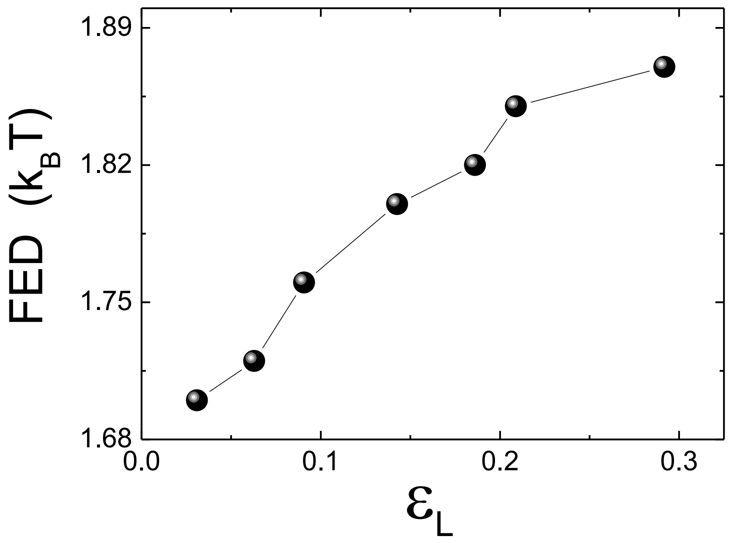

Figure S9. The free energy difference between the minima and maxima as a function of the strain along longitudinal direction.

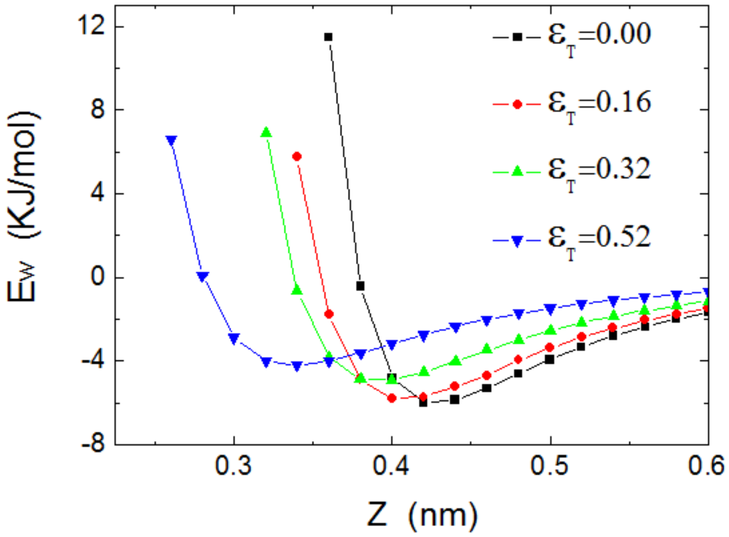

Figure S10. The energy profile of single water molecule on phosphorene surface with various strain as a function of distance from surface.

References:

1. Malani, A. & Ayappa, K.G. Relaxation and jump dynamics of water at the mica interface. *The Journal of chemical physics* 136, 194701 (2012).
